# Real-time Structural Tracking of Slow P-type ATPase Dynamics

**DOI:** 10.1007/s00232-026-00389-0

**Published:** 2026-08-11

**Authors:** K. Magkakis, F. Sabzian-Molaei, F. Orädd, T. Plivelic, M. Andersson

**Affiliations:** 1 Department of Chemistry, Umeå University, Umeå, Sweden; 2 MAX IV Laboratory, Lund University, Lund, Sweden

**Keywords:** P-type ATPase,, Membrane protein, Time-resolved X-ray solution scattering

## Abstract

Time-resolved X-ray solution scattering (TR-XSS) provides direct access to protein structural dynamics but has largely been restricted from the microsecond range up to approximately 100 milliseconds. As a result, slower enzymatic systems, including many P-type ATPases, remain difficult to probe. Here, we extend the temporal reach of TR-XSS by sequentially positioning radiation damage-free acquisition windows to enable capturing structural evolution across sub-second to second timescales. Implemented at the CoSAXS beamline at MAX IV Laboratory, this strategy enables continuous tracking of slow protein dynamics while preserving structural sensitivity. Using adenylate kinase (AdK) as a benchmark, we observed a single conformational transition accompanied by signal amplitude decay. In contrast, application to the prokaryotic P-type ATPase LMCA1 revealed clear evolution in scattering profiles, consistent with sequential conformational transitions. Kinetic analysis identified two transitions on the 140 ms and 660 ms timescales, which correspond monitoring rise and decay of a rate-limiting step which can symbolize intermediate dynamics in a slow transport cycle. The results demonstrate that extended-time TR-XSS can resolve multi-step reaction pathways in slow membrane proteins. The approach broadens the accessible timescale of TR-XSS and establishes a general framework for studying slow conformational dynamics in P-type ATPases and related systems.

**Graphical Abstract:**

**Figure:**
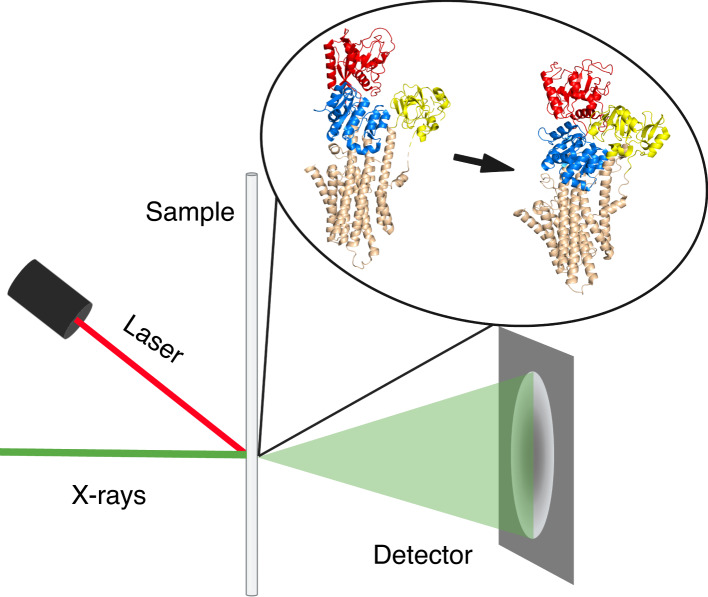

**Supplementary Information:**

The online version contains supplementary material available at 10.1007/s00232-026-00389-0.

## Introduction

Conformational dynamics is at the heart of P-type ATPase function and enable the ATP-driven transport of a wide range of substrates across biological membranes. While high-resolution structural methods have provided detailed snapshots of key intermediates, understanding how these states are connected in time remains a central challenge in the field. Synchrotron-based time-resolved X-ray solution scattering (TR-XSS) has emerged from early grazing x-ray incidence diffraction studies (Blasie et al. 1985) into a powerful tool to monitor real-time P-type ATPase dynamics (Orädd et al. 2021, Ravishankar et al. 2020, Sabzian-Molaei et al. 2026), in addition to light-sensitive proteins (Andersson et al. 2009, Malmerberg et al. 2011) and chemicals (Vincent et al. 2009, Georgiou et al. 2006). However, the method is intrinsically best suited to track timescales from the microsecond range up to approximately 100 milliseconds, where high repetition rates enable robust signal detection. As a consequence, TR-XSS studies have exclusively focused on P2A-subtype transporters (Ravishankar et al. 2020, Prabudiansyah et al. 2024), which exhibit turnover rates in the range of ~ 5–100 s^-1^(Dode L et al. 2002, Dyla M et al. 2017) However, P-type ATPase transport spans a wide temporal regime with P1B-subtype heavy-metal transporters and P4-subtype lipid flippases being considerably slower, often functioning on sub-second to second timescales (Arguello et al. 2003, Wang et al. 2014, Ravishankar et al. 2018, Zhou et al. 2009). Subtypes P3 and P5 ATPases are less well characterized structurally, but are also generally found to engage in slow transport (Serrano 1983, McKenna et al. 2020, Sorensen et al. 2015). This highlights the need for experimental strategies capable of extending TR-XSS measurements into longer timescales while maintaining structural sensitivity.

One promising strategy to extending TR-XSS to longer timescales is to exploit fast detector readout to continuously monitor structural changes over extended time periods with successive replenishments of the sample (Westenhoff et al. 2010). However, continuous X-ray exposure raises concerns regarding radiation damage, which can obscure subtle conformational signals and complicate data interpretation. We recently demonstrated that, under defined experimental conditions, no discernible radiation damage occurs within a 50 ms temporal window (Magkakis et al. 2024). This observation suggests a strategy in which such X-ray damage-free windows can be sequentially positioned across longer timescales, spanning hundreds of milliseconds to seconds, and subsequently combined to reconstruct the full time evolution of the system. Such a TR-XSS strategy could be key to time-resolved characterization of slow subtypes in the P-type ATPase family.

Here, we implement this approach at a the multi-purpose SAXS beamline CoSAXS at the MAX IV Laboratory synchrotron and evaluate its performance by tracking structural dynamics in the adenylate kinase enzymatic reaction and of the prokaryotic calcium-transporting P-type ATPase from *Listeria monocytogenes* (LMCA1) (Hansen et al. 2021). By comparing a system with largely two-state behavior to one requiring multiple intermediates, we establish the capability of this method to resolve both kinetic decay and conformational transitions across extended timescales.

## Results and Discussion

### The Experimental Design

**Fig. 1:**
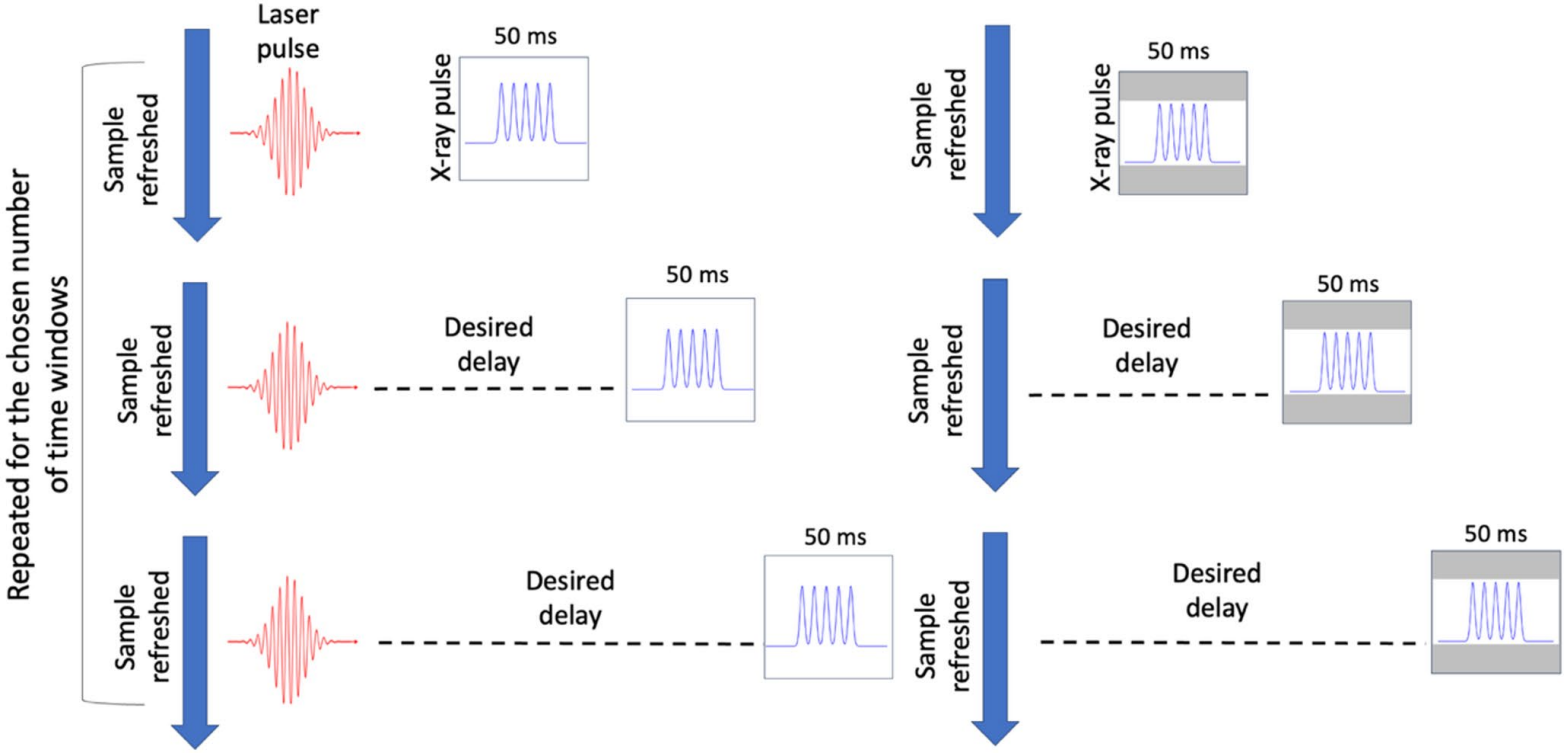
Experimental strategy. Laser-excited (left) and dark (right) experiments using a radiation damage-free 50-ms temporal window of X-ray exposure with the sample being refreshed after each such exposure. The X-ray exposure is delayed for defined time periods after time zero (laser excitation) to probe slow conformational dynamics

Time-resolved X-ray solution scattering (TR-XSS) experiments were performed at the CoSAXS beamline at MAX IV Laboratory, Sweden, using 12.4 keV X-rays (Herranz-Trillo et al. 2024). Scattering data were recorded in the range ~ 0.01–0.6 Å^-1^ with millisecond time resolution set by the detector frame rate (2 ms per frame). Samples were delivered in a capillary flow cell and refreshed between measurements to avoid radiation damage. The ATP-dependent reactions were initiated by nanosecond laser photolysis of NPE-caged ATP (355 nm) (McCray et al. 1980). The laser spot exceeded the X-ray beam size to ensure homogeneous activation within the X-ray probe volume. Laser excitation and X-ray acquisition were synchronized through a TANGO-based control system (Silva et al. 2023). Defined delay times were introduced subsequent to laser onset to enable detecting longer timepoints while minimizing X-ray radiation damage (Fig. 1). Data were collected in alternating laser-on and laser-off cycles to avoid experimental drift and background effects.

### Equilibrium Measurements of the Adenylate Kinase Benchmark System

**Fig. 2:**
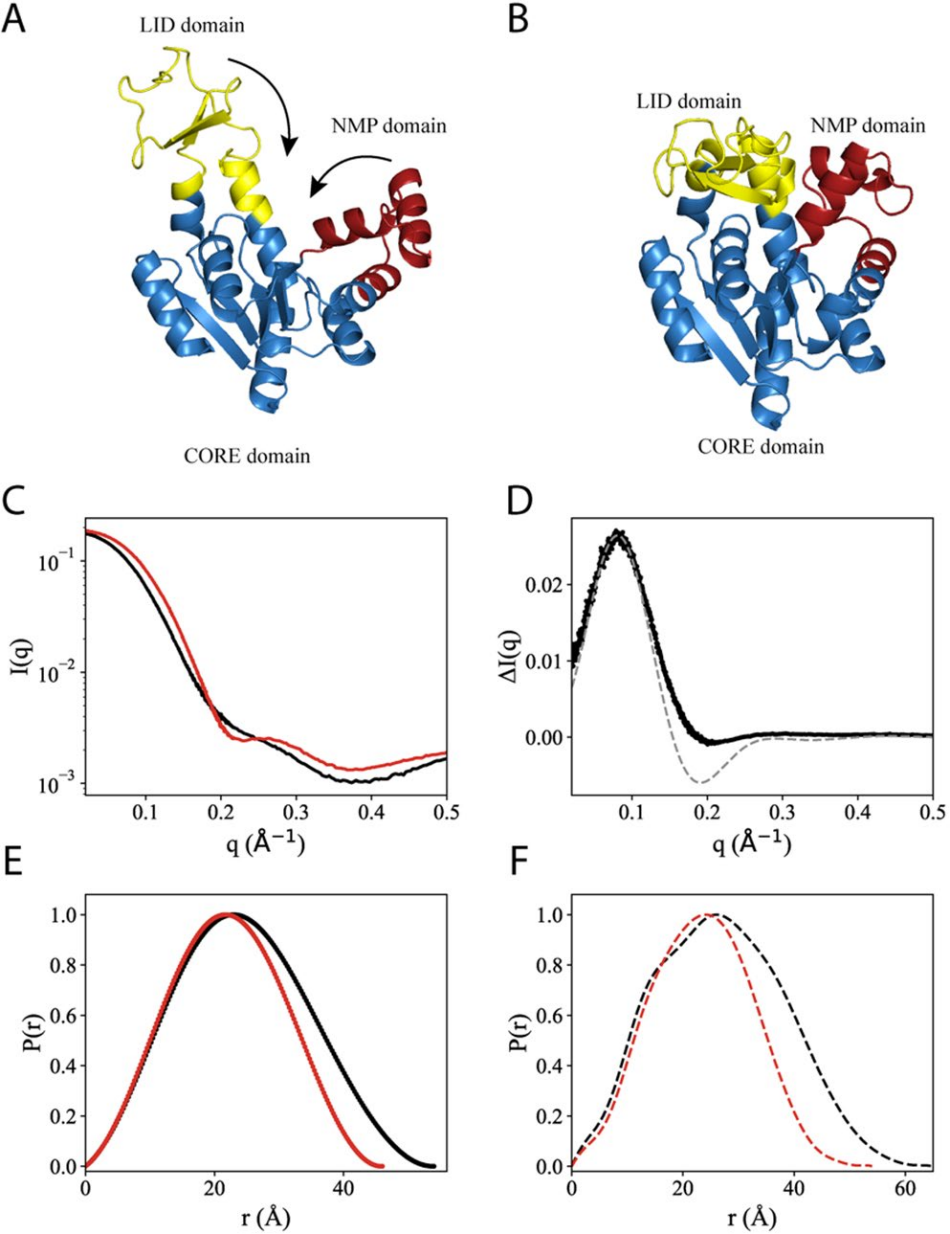
Adenylate kinase crystal and solution structures at 12.5 mg/mL protein concentration. Crystal structures of the **(A)** open and **(B)** closed conformations with the ATP-binding (LID, in yellow), AMP-binding (NMP, red), and CORE (blue) domains. **(C)** Small/wide-angle X-ray scattering data in absence (black) and presence (red) of ATP. **(D)** Difference X-ray scattering profile obtained from subtracting scattering without ATP from that with ATP for the experimental (black) and theoretical (dashed, gray) curves. Pair distance distribution (P(r)) functions obtained from measurements without ATP (black) and with ATP (red) from the **(E)** experimental data and **(F)** calculated theoretical curves

Adenylate kinase (AdK) is a 23 kDa soluble protein that catalyses interconversion of nucleotides by phosphoryl transfer (Dzeja et al. 2009). The AdK enzyme is therefore central to regulation of cellular energy levels, but also a well-exploited model system in methods development aimed at tracking protein conformational changes (Song et al. 2013, Nam et al. 2024, Wang et al. 2013, Li et al. 2015). Upon ATP release in the presence of AMP, the AdK enzyme will relocate its ATP-binding (LID) and AMP-binding (NMP) domains from open configurations towards closed, and catalytically active, states with both domains attached to the central, relatively immobile CORE domain. The extent of this conformational change can be visualized by open (Fig. 2A) and closed (Fig. 2B) crystal structures (Muller et al. 1996, Muller et al. 1992). However, it is known from NMR experiments that AdK will display a wide range of domain configurations in the absence of ATP substrate (Hanson et al. 2007). Before tracking the enzyme in real time, we therefore first performed static small-angle X-ray scattering experiments with and without ATP. These equilibrium measurements were conducted at two different protein concentrations, 2.5 mg/mL and 12.5 mg/mL.

The obtained scattering profiles showed significant differences between the ATP conditions (Fig. 2C). Because the observed differences were similar but not as pronounced at 2.5 mg/mL protein concentration due to lower signal-to-noise (Suppl. Figure 1), we decided to proceed with analysing the more (12.5 mg/mL) concentrated sample to align better with the subsequent TR-XSS measurements. We then calculated a difference scattering profile by subtracting the scattering profile obtained in the absence of ATP from that obtained with ATP present in the solution (Fig. 2D). The difference profile showed a strong positive peak centered around 0.1 Å^−1^ with a negative feature at 0.2 Å^−1^. These features were similar to those observed in the theoretical difference profile obtained from the crystal structures. The enhanced negative feature at 0.2 Å^−1^ reflects that the theoretical difference profile is calculated from crystal structures corresponding to discrete endpoint conformations, whereas the experimental SAXS curves represent ensemble-averaged solution states. The smaller experimental differences are therefore consistent with ligand-induced population shifts within a dynamic conformational ensemble rather than complete conversion between two crystal structures.

In addition, the experimentally obtained pair distance distribution P(r) curves showed a significantly more closed AdK conformation when ATP was present (Fig. 2E). Theoretical scattering profiles calculated from the open and closed crystal structure showed very similar trends (Fig. 2F). Therefore, while NMR experiments have shown that the AdK free energy landscape is relatively flat in the absence of ATP and can visit a wide ensemble of structures (Hanson et al. 2007), at equilibrium the two conditions (with and without ATP) are explained well by the domain configurations in the crystal structures. Knowing the expected difference curve shape associated with a catalytically active form of the AdK enzyme, we could now perform time-resolved experiments to track this reaction over an extended time.

### Tracking the AdK Enzymatic Catalysis Over Time

**Fig. 3:**
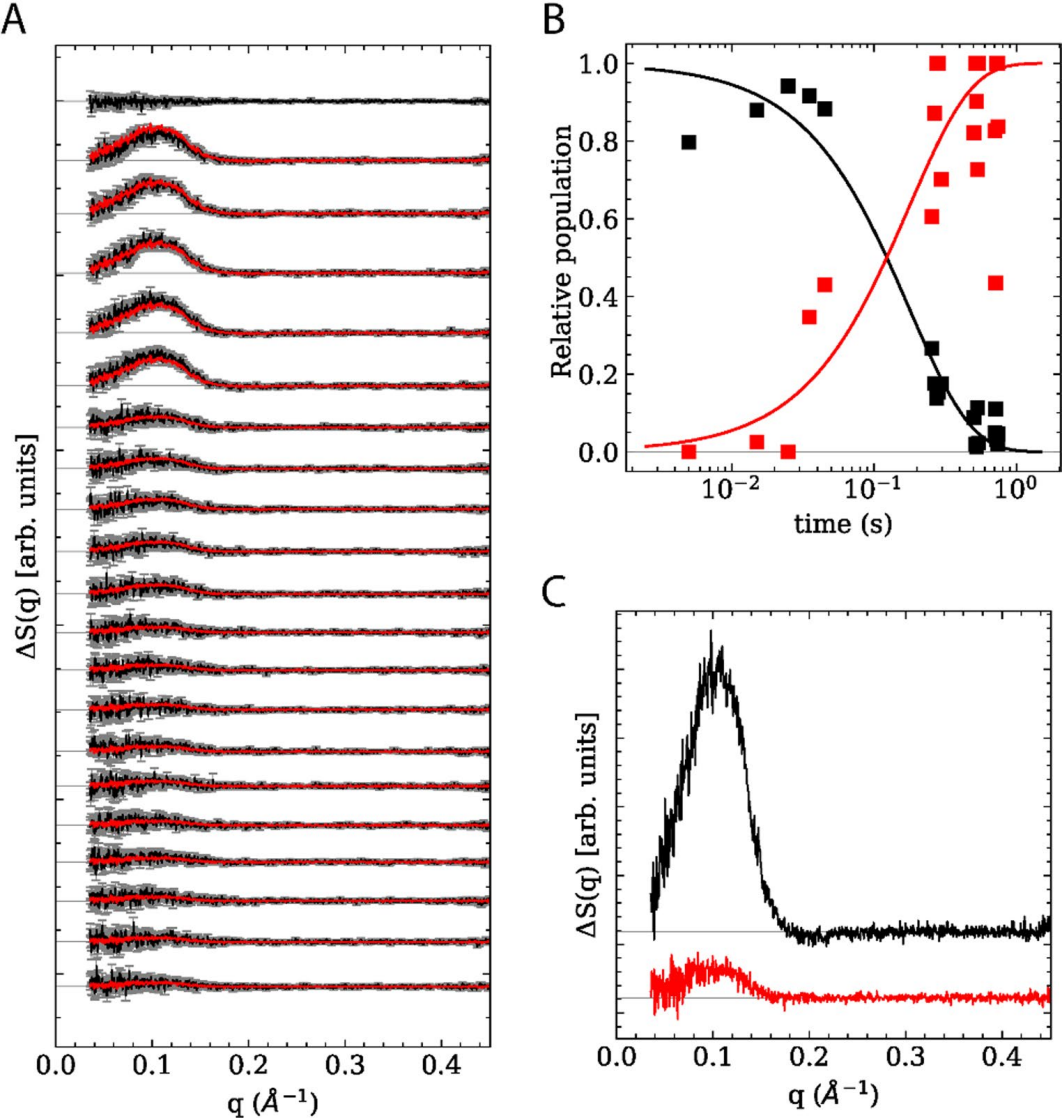
Time-resolved measurement of the adenylate kinase reaction at 27 mg/mL protein concentration. **(A)** Time-resolved data (black) and reconstituted data from the two-state kinetic model (red). **(B)** Temporal evolution of the populations of the early (black) and late (red) modelled states. **(C)** The corresponding time-independent early (black) and late (red) basis spectra

Time-resolved experiments were triggered by laser-induced ATP release from a NPE photosensitive cage. The AdK reaction dynamics were monitored in four 50-ms windows distributed across 0–50 ms, 250–300 ms, 500–550 ms, and 700–750 ms, with 5 measurements in each such window. During the observed 750 ms, a signature difference scattering signal developed within the first 0–50 ms that consisted of a positive peak centered around 0.1 Å^-1^ and a negative, less pronounced feature at 0.2 Å^-1^ (Fig. 3A). The observed difference signal closely resembled the equilibrium difference profile associated with the open-to-closed transition (Fig. 2D). This signal developed in the 0–50 ms window and persisted into 250–300 ms after which it gradually decayed on the longer timescales.

**Fig. 4:**
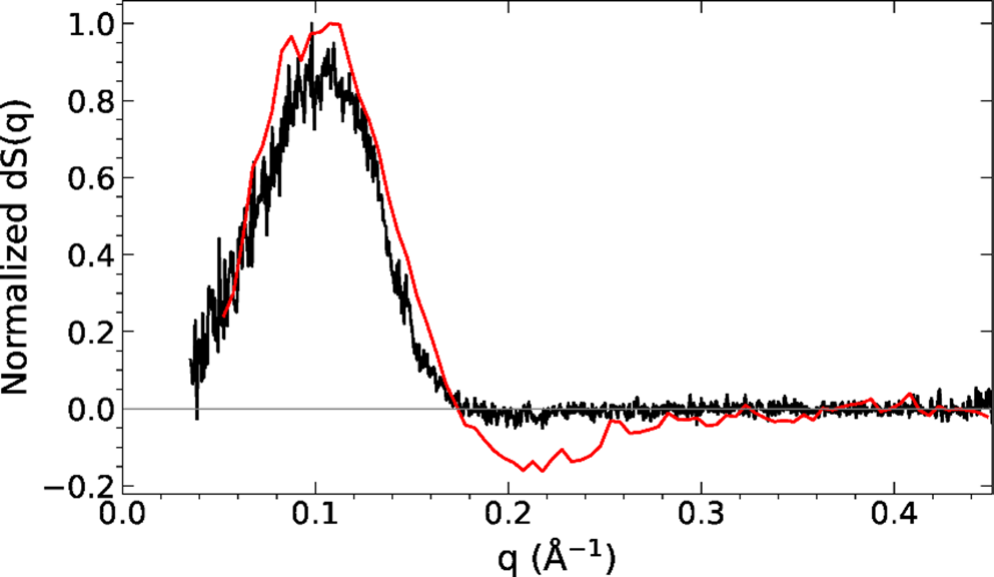
Comparison of AdK dynamics monitored at a dedicated time-resolved synchrotron beamline aimed at faster dynamics and a multi-purpose SAXS beamline with fast detector readout (Magkakis et al. 2025). The late basis spectrum showing faster AdK dynamics (red) shown together with the early (black) basis spectrum from the current slow measurements. The similar overall shape of the spectra indicates that both experiments monitor related AdK conformational changes, although the kinetic components correspond to different stages of the reaction

Previous TR-XSS studies of AdK have commonly used sequential two-state kinetic models to describe the time-resolved data (Magkakis et al. 2024, Orädd et al. 2021, Magkakis et al. 2025). We therefore tested such a model for the present dataset and obtained a reasonable reconstruction of the experimental signal (χ^2^ = 1.14) (Fig. 3A, B). The corresponding early and late time-independent basis spectra showed similar overall profiles and differed mainly in amplitude (Fig. 3C). This behavior is consistent with a dominant structural response in which the time dependence reflects changes in population within a conformational ensemble, rather than the appearance of distinct structural intermediates. The slow component obtained from the present data was ~ 180 ms. This value should not be directly compared with the 32 ms time constant reported in the previous TR-XSS experiment at beamline ID09 at ESRF, where formation of an intermediate AdK state was resolved (Magkakis et al. 2025). In the present experiment, formation of this state is not resolved. Instead, the dominant feature is the decay of the time-resolved SAXS signal, described by a slow relaxation component of ~ 180 ms. Since the AdK reaction is reversible, we interpret this decay as progressive loss of synchronization of the initially triggered enzyme population as the molecules proceed through forward and reverse catalytic steps and relaxation into equilibrium. Importantly, the basis spectrum associated with this decay has a similar overall shape to the previously reported AdK difference signal (Fig. 4), supporting that both experiments monitor related AdK conformational changes, although at different stages of the reaction.

We also monitored AdK dynamics up to 1.6 s. This dataset had lower signal-to-noise because of suboptimal laser characteristics and therefore resulted in less well-modelled conformational transitions (Suppl. Figure 2). Nevertheless, it showed the same overall relaxation trend, which supports that the slow component observed reflects AdK conformational relaxation rather than a separate structural process.

In contrast to AdK, P-type ATPases such as LMCA1 initiate transport from a relatively well-defined ground state and proceed through a sequence of structurally distinct intermediates (Prabudiansyah et al. 2024, Dyla et al. 2017, Hansen et al. 2021, Basse Hansen et al. 2025). Consequently, on long timescales, P-type ATPase time-resolved scattering signals are expected to reflect genuine conformational transitions between discrete states rather than simple amplitude changes of a single structural difference.

### Tracking Slow Conformational Dynamics in the LMCA1 Reaction

**Fig. 5:**
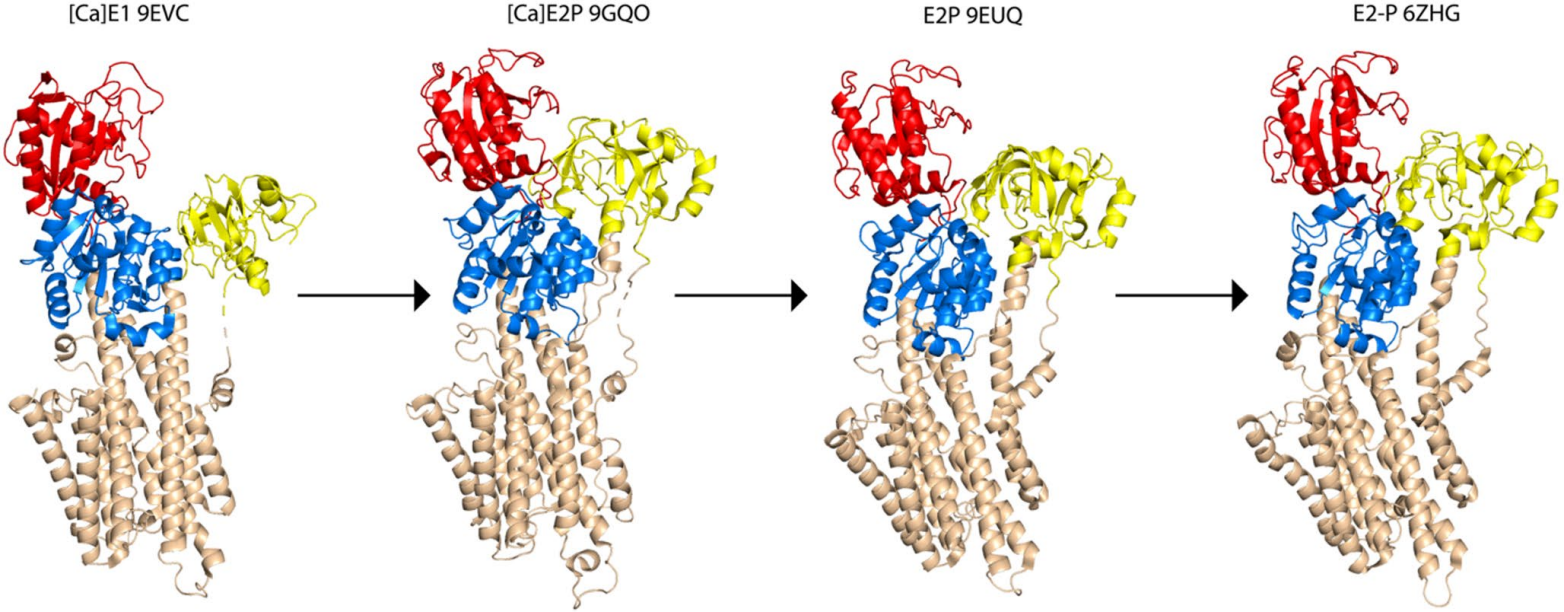
LMCA1 sequential dynamics displayed by structures trapped in [Ca]E1, [Ca]E2P, E2P, and E2-P states with A- (yellow), N- (red), P- (blue), and M-domain (light brown)

For LMCA1, the reaction cycle begins by ATP binding to a calcium-bound state [Ca]E1 that undergoes phosphorylation into an E1P state, which marks the start of the transition towards the low affinity E2 states and ion release. Dephosphorylation brings the protein back to the starting E1 configuration. A few principal intermediates have been structurally characterized, including a calcium-bound E1 state (Prabudiansyah et al. 2024) (PDB ID: 9EVC), a four-glycine mutant trapped in a calcium-bound E2P state (Basse Hansen et al. 2025) (PDB ID: 9GQO), and E2 states associated with dephosphorylation (Prabudiansyah et al. 2024, Hansen et al. 2021) (PDB ID: 9EUQ and 6ZHG) (Fig.5). Hence, P-type ATPases, in contrast to adenylate kinase, proceed through a series of structurally distinct intermediates during turnover. We therefore next applied the extended-time TR-XSS strategy to LMCA1 to test whether the method could resolve not only signal decay, but also sequential conformational transitions in a slow P-type ATPase reaction.

**Fig. 6:**
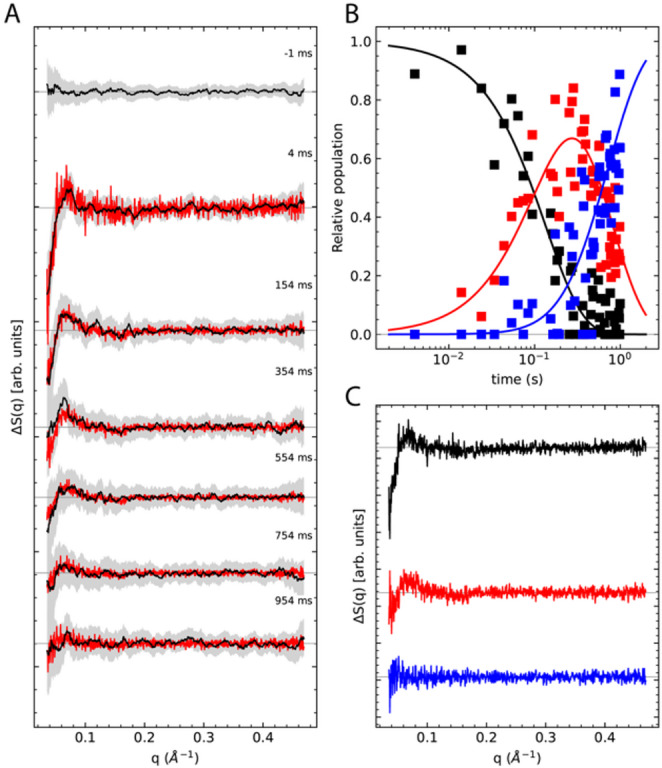
Time-resolved measurement of the LMCA1 P-type ATPase reaction at 15 mg/mL protein concentration. **(A)** Time-resolved data (black, running average) with error bars (gray) and reconstituted data from a three-state kinetic model (red) shown for selected timepoints for clarity. **(B)** Temporal evolution of the populations of the early (black), intermediate (red), and late (blue) modelled states for all 55 timepoints. **(C)** The corresponding time-independent early (black), intermediate (red), and late (blue) basis spectra

The reaction was again initiated by photorelease of ATP from NPE-caged ATP, and scattering data were collected in eleven 50-ms windows of 0–50 ms, 50–100 ms, 150–200 ms, 250–300 ms, 350–400 ms, 450–500 ms, 550–600 ms, 650–700 ms, 750–800 ms, 850–900 ms, and 950–1000 ms. During this total time of 1 s, the LMCA1 time-resolved scattering data showed a clear evolution in signal shape across the measured time range, which could not be explained by a simple amplitude change (Fig. 6A). Accordingly, sequential kinetic models converged on an initial early-to-intermediate transition followed by a substantially slower intermediate-to-late transition, with transition times of 140 ms and 660 ms, respectively (Fig. 6B).

**Fig. 7:**
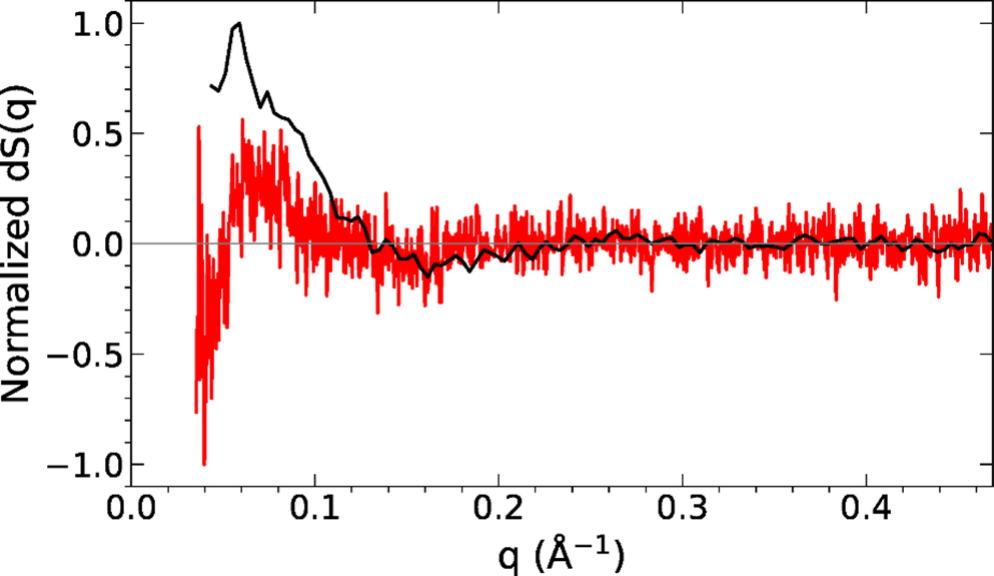
Comparison of LMCA1 dynamics monitored at a dedicated time-resolved synchrotron beamline aimed at faster dynamics (Prabudiansyah et al. 2024) and a multi-purpose SAXS beamline with fast detector readout. The late basis spectrum showing faster LMCA1 dynamics (black) together with the intermediate basis spectrum (red) from the current slow measurements

Interpreting the time-resolved data as a multi-state reaction sequence was further supported by the time-independent basis spectra. In contrast to the AdK dataset, the LMCA1 basis spectra showed distinct profiles, consistent with structural changes between discrete conformational states (Fig. 6C). This behavior agrees with the expected reaction landscape of a P-type ATPase, which starts from a comparatively well-defined ground state and progresses through a sequence of intermediates during ATP-driven transport. The early-to-intermediate transition corresponds to conversion from a pre-pulse equilibrium state into the previously observed phosphorylation-associated, rate-limiting step in the LMCA1 reaction (Prabudiansyah et al. 2024). Indeed, the corresponding basis spectra match qualitatively (Fig. 7). Because the previously published 10.4 ms transition time were collected at a dedicated time-resolved beamline optimized for faster timepoints, we here report a longer transition time than before. This correlates with a formation time of 77 ms for the LMCA1 observed by [γ-³²P]ATP phosphorylation (Dyla et al. 2017), given that we monitored at 20 °C compared to 25 °C in the reported experiment. However, it is clear that a 3-state kinetic model best explains the data and therefore the design presented here can capture similar dynamics and track for an extended time.

The intermediate-to-late transition extends into the few-hundred-millisecond range characteristic of slow catalytic steps in P-type ATPases. In the LMCA1 case, this represents tracking the rate-limiting step over time. Hence, its depletion corresponds to ATP being hydrolyzed in the sample and in contrast to AdK, LMCA1 cannot reversibly regenerate ATP under these conditions. Importantly, the ability to resolve both transitions within a single reconstructed time series demonstrates that sequential placement of radiation-safe acquisition windows can extend TR-XSS into a temporal regime relevant for slow membrane transporters. In this sense, LMCA1 provides a proof of principle that the approach is suitable for following slow enzymatic conformational dynamics in systems that cannot be captured within conventional micro- to millisecond experiments.

Together, these results establish that this fast detector-readout strategy can recover time-resolved structural information from a slow P-type ATPase reaction over the seconds timescale, and can distinguish multi-step conformational transitions from simple signal decay. This considerably extends the temporal scope of TR-XSS from studies of micro- to 100 milliseconds dynamics (Orädd et al. 2021, Andersson et al. 2009, Malmerberg et al. 2011, Berntsson et al. 2019, Berntsson et al. 2017) of protein dynamics to include slow P-type ATPase membrane transport, e.g. copper and zink transport (Wang et al. 2014, Salustros et al. 2022, Gronberg et al. 2021, Gronberg et al. 2016, Andersson et al. 2014) and regulation (Guo et al. 2024, Orädd et al. 2022).

## Methods

### Data Acquisition

All data collections were carried out at the CoSAXS beamline at the MAXIV Laboratory synchrotron, with a 12.4 keV a monochromatic X-ray beam and controlled 20±0.5 °C temperature. In the SAXS measurements, an Eiger 2 detector at 3.5 m sample-to-detector distance registered data up to 0.3 Å^−1^ and a Pilatus detector at 0.5 m from the sample covered until 3.0 Å^−1^. Two protein concentrations were used, 2.5 mg/ml and 12.5 mg/ml. The sample was exposed for 20 ms at a time at continuous flow and 1,000 data points were collected along the capillary and Correlation Map was used to select reproducible points (Franke et al. 2015).

For the time-resolved measurements, the Eiger 2 detector was placed at 2.8 m from the sample covering up to 0.5 Å^−1^, and a Mythen detector at 0.25 m. The laser power was adjusted at 1.06 kV with a fluence of about 5.2 mJ/mm^2^. The samples were successively replenished within a closed delivery circuit through a vertically positioned 0.3 mm quartz capillary with a peristaltic pump operating in discontinuous mode.

For the AdK measurements, the sample was prepared at 27 mg/mL with 10 mM of NPE-caged ATP added just before the measurements, as described previously (Orädd et al. 2021). Time blocks of 0–50 ms, 250–300 ms, 500–550 ms and 700–750 ms were each monitored for 1,000 repetitions, with 5 measurements in each such window. For the LMCA1 measurements, the protein was prepared at 15 mg/mL with 10 mM of NPE-caged ATP added just before the measurements and the time blocks of 0–50 ms, 50–100 ms, 150–200 ms, 250–300 ms, 350–400 ms, 450–500 ms, 550–600 ms, 650–700 ms, 750–800 ms, 850–900 ms, 950–1000 ms were each monitored for 456 repeats.

### Data Analysis

For the AdK SAXS data, buffer subtraction further analysis was carried out using the ATSAS software (Manalastas-Cantos et al. 2021). The difference curves were calculated based on merged absolute curves from both the Eiger and Pilatus detectors. For the pair-distance distribution analysis, the non-merged datasets derived from the Eiger detector were used.

For the time-resolved data, after azimuthal radial integration of the detector images to obtain the absolute scattering profiles, the data were normalised in the 0.35 Å^−1^ < q < 0.45 Å^−1^ region where scattering changes were minimal. After normalization, frames were grouped according to their recorded acquisition delay. Difference scattering curves were then calculated as:

$$\:\varDelta\:{S}_{j}\left(q\right)={I}_{\mathrm{l}\mathrm{i}\mathrm{g}\mathrm{h}\mathrm{t},j}\left(q\right)-{I}_{\mathrm{d}\mathrm{a}\mathrm{r}\mathrm{k},j}\left(q\right)$$

for each paired observation * j*.

For a given delay, the mean difference spectrum was calculated across all paired observations as:

$$\:\overline{\varDelta\:S}\left(q\right)=\frac{1}{N}\sum\:_{j=1}^{N}\varDelta\:{S}_{j}\left(q\right)$$

where * N* is the number of paired light-dark differences available for that delay.

The uncertainty was estimated as the standard error of the mean (SEM), using the sample standard deviation with * N-1* degrees of freedom:

$$\:\mathrm{S}\mathrm{E}\mathrm{M}\left(q\right)=\frac{s\left(q\right)}{\sqrt{N}}$$

where $\:s\left(q\right)$ is the sample standard deviation calculated across the set of delay-specific difference curves. To reduce noise and compress the time series into broader temporal blocks, adjacent delay-resolved spectra were merged in groups of five (10 ms groups). For each merge block, the mean spectrum was calculated as

$$\:{\overline{\varDelta\:S}}_{\mathrm{m}\mathrm{e}\mathrm{r}\mathrm{g}\mathrm{e}}\left(q\right)=\frac{1}{M}\sum\:_{k=1}^{M}{\overline{\varDelta\:S}}_{k}\left(q\right)$$

where * M* is the number of spectra in the block.

### Kinetic Modelling

For the kinetic modelling, the AdK data were described using a sequential 2-state model. Integrated first-order rate equations were used to describe the model as follows:

$$\:Early\:\left(t\right)\:=\:{e}^{-{k}_{1}t}$$

$$\:Late\:\left(t\right)\:=\:1\:-\:{e}^{-{k}_{1}t}$$

where k1 is the rate constant that controls the decay of the early state.

For the LMCA1 data, a 3-state sequential model was used to describe the sequential conversion from an initial early state to an intermediate state, followed by conversion to a final late state. In closed form, the first population is:

$$\:Early\:\left(t\right)={e}^{-t/{k}_{1}}$$

and for $\:{k}_{1}\ne\:{k}_{2}$ the intermediate population is

$$\:Intermediate\:\left(t\right)=\frac{{k}_{2}}{{k}_{2}-{k}_{1}}\left({e}^{-t/{k}_{2}}-{e}^{-t/{k}_{1}}\right)$$

while the late population can be written as

$$\:Late\:\left(t\right)=1-A\left(t\right)-B\left(t\right)$$

## Supplementary Information

Below is the link to the electronic supplementary material.

Supplementary Material 1

## Data Availability

We will share data upon publication.
