# Supplementary material for: Real-time Structural Tracking of Slow P-type ATPase Dynamics
Source: J Membr Biol. 2026 Aug 11;259(1):28. doi: 10.1007/s00232-026-00389-0 (PMC13461796; doi:10.1007/s00232-026-00389-0)
Supplement: Supplementary file 1 [file 232_2026_389_MOESM1_ESM.docx]

**Supplementary Information**

**Real-time structural tracking of slow P-type ATPase dynamics**

Magkakis, K.^a^, Sabzian-Molaei, F.^a^, Orädd, F. ^a^, Plivelic, T.^b^, Andersson, M.^a,*^

^a^ Department of Chemistry, Umeå University, Umeå, Sweden

^b^ MAX IV Laboratory, Lund University, Lund, Sweden

^*^ Correspondence e-mail: magnus.p.andersson@umu.se

**Supplementary Figure 1.** Small/wide-angle X-ray scattering data of adenylate kinase at 2.5 mg/mL protein concentration in absence (black) and presence (red) of ATP.

**Supplementary Figure 2.** Time-resolved measurement of the adenylate kinase reaction. **(A)** Time-resolved data (black) and reconstituted data from the two-state kinetic model (red) monitored in seven 50-ms windows distributed across 0-50 ms, 50-100 ms, 350-400 ms, 650-700 ms, 950-1000 ms, 1250-1300 ms, and 1550-1600 ms. **(B)** Temporal evolution of the populations of the early (black) and late (red) modelled states. **(C)** The corresponding time-independent early (black) and late (red) basis spectra.
